# Coordinated Formation of IMPDH2 Cytoophidium in Mouse Oocytes and Granulosa Cells

**DOI:** 10.3389/fcell.2021.690536

**Published:** 2021-05-28

**Authors:** Shiwen Ni, Teng Zhang, Chenmin Zhou, Min Long, Xuan Hou, Liji You, Hui Li, Lanying Shi, You-Qiang Su

**Affiliations:** ^1^State Key Laboratory of Reproductive Medicine, Nanjing Medical University, Nanjing, China; ^2^Shandong Provincial Key Laboratory of Animal Cells and Developmental Biology, School of Life Sciences, Shandong University, Qingdao, China; ^3^Women’s Hospital of Nanjing Medical University, Nanjing Maternity and Child Health Hospital, Nanjing Medical University, Nanjing, China; ^4^Collaborative Innovation Center of Genetics and Development, Fudan University, Shanghai, China

**Keywords:** IMPDH2, cytoophidium, oocyte maturation, granulosa cell, mycophenolic acid, guanine nucleotide, female fertility

## Abstract

Inosine monophosphate dehydrogenase (IMPDH), the rate-limiting enzyme catalyzing *de novo* biosynthesis of guanine nucleotides, aggregates under certain circumstances into a type of non-membranous filamentous macrostructure termed “cytoophidium” or “rod and ring” in several types of cells. However, the biological significance and underlying mechanism of IMPDH assembling into cytoophidium remain elusive. In mouse ovaries, IMPDH is reported to be crucial for the maintenance of oocyte–follicle developmental synchrony by providing GTP substrate for granulosa cell natriuretic peptide C/natriuretic peptide receptor 2 (NPPC/NPR2) system to produce cGMP for sustaining oocyte meiotic arrest. Oocytes and the associated somatic cells in the ovary hence render an exciting model system for exploring the functional significance of formation of IMPDH cytoophidium within the cell. We report here that IMPDH2 cytoophidium forms *in vivo* in the growing oocytes naturally and *in vitro* in the cumulus-enclosed oocytes treated with IMPDH inhibitor mycophenolic acid (MPA). Inhibition of IMPDH activity in oocytes and preimplantation embryos compromises oocyte meiotic and developmental competences and the development of embryos beyond the 4-cell stage, respectively. IMPDH cytoopidium also forms *in vivo* in the granulosa cells of the preovulatory follicles after the surge of luteinizing hormone (LH), which coincides with the resumption of oocyte meiosis and the reduction of IMPDH2 protein expression. In cultured COCs, MPA-treatment causes the simultaneous formation of IMPDH cytoopidium in cumulus cells and the resumption of meiosis in oocytes, which is mediated by the MTOR pathway and is prevented by guanosine supplementation. Therefore, our results indicate that cytoophidia do form in the oocytes and granulosa cells at particular stages of development, which may contribute to the oocyte acquisition of meiotic and developmental competences and the induction of meiosis re-initiation by the LH surge, respectively.

## Introduction

Meiosis is a type of specialized cell division through which germ cells reduce their chromosome composition from diploid to haploid. Faithful completion of the first meiosis is therefore a critical step toward producing a functionally normal gamete. In females of most mammalian species, meiosis starts in the fetal ovaries and stops at diplotene stage around the birth. Meiosis in oocyte is then arrested at this particular stage of prophase I (also referred to as dictyate stage) during the entire phases of oocyte and follicle growth, and won’t resume until the emergence of the surge of preovulatory luteinizing hormone (LH) at puberty or adulthood. After the LH surge, meiosis resumes in the fully grown oocyte (FGO) of preovulatory follicles, which is manifested by the morphological changes in the nucleus, i.e., breakdown of the germinal vesicle (also commonly known as GVB or GVBD), and enters the M-phase. The first meiotic division is ended with the equal distribution of a set of haploid homologous chromosomes into the asymmetrically divided large secondary oocyte and small first polar body (PB1). The resultant mature oocyte with meiosis arrested at metaphase II, which is now referred to as egg, is ovulated together with the mucified cumuli oophori into the oviduct ready for fertilization (Eppig et al., 2004; Conti and Franciosi, 2018).

Oocytes acquire the competence to resume and complete the first meiosis in a stepwise manner during the growing process. They first become competent to undergo GVBD in the small antral follicles, and then gain full competence to mature to M II when they reached the fully grown stage in the large antral follicles (Eppig et al., 2004). Such that, as demonstrated in the classic experiment by Pincus and Enzmann in the 1935 (Pincus and Enzmann, 1935), fully grown oocytes are readily to undergo GVBD and extrude PB1 (a.k.a. meiotic maturation) spontaneously when they are liberated from the large antral follicles before the LH surge and cultured in suitable medium. However, meiotic maturation cannot occur spontaneously *in vivo* in the FGO, hence, a factor (or factors) was then postulated to be present within the microenvironment of the antral follicles to prevent the FGOs from reentering into meiosis (Pincus and Enzmann, 1935; Tsafriri et al., 1976). Although this postulation on oocyte meiotic arrest was made nearly a century ago, landmark discoveries on the identity, origin, and functioning mechanisms of the putative oocyte maturation inhibitory factor (s) were achieved only in the past one decade or so. It was found that natriuretic peptide type C (NPPC) secreted by mural granulosa cells (MGCs) and its cognate guanylyl cyclase-linked natriuretic peptide receptor 2 (NPR2) expressed by cumulus cells maintain oocyte meiotic arrest (Zhang et al., 2010). This particular combination of natriuretic peptide system induces the production of cGMP in cumulus cells, which in turn diffuses into the oocyte through the heterologous gap junctions connecting them and suppresses the cAMP-hydrolysising activity of PDE3A. High levels of cAMP are then sustained in oocytes, which subsequently maintain the oocyte at meiotic arrest (Norris et al., 2009; Vaccari et al., 2009).

**FIGURE 1:**
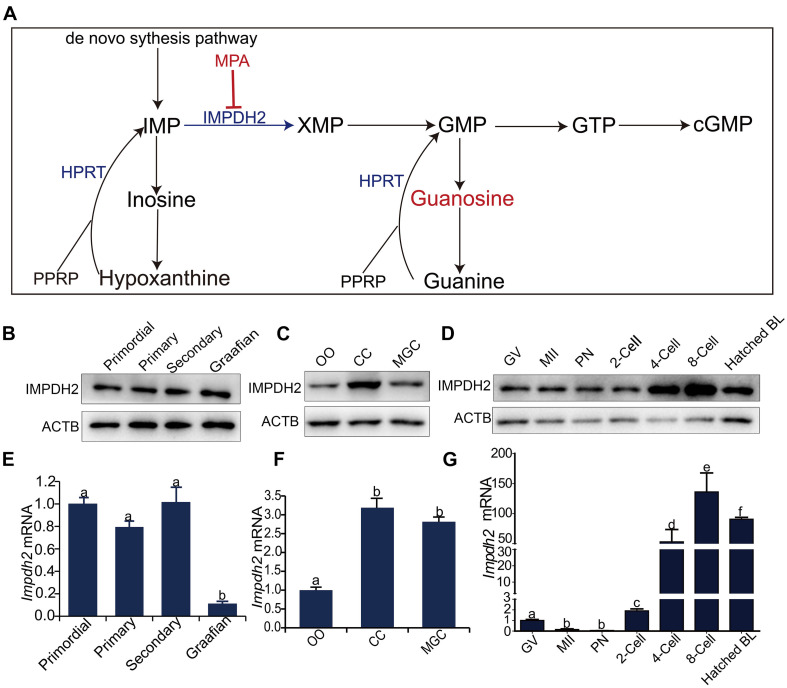
Dynamic expression of *Impdh2* in oocytes, granulosa cells, and preimplantation embryos. **(A)** Schematic illustration of the guanosine nucleotides biosynthesis. **(B–D)** WB detection of the expression of IMPDH2 protein in oocytes isolated from the primordial, primary, secondary, and Graafian follicles **(B)**; oocytes (OO), cumulus cells (CC), and mural granulosa cells (MGC) isolated from the large antral follicles **(C)**; oocytes at GV- and MII stages, and preimplantation embryos at different developmental stages **(D)**. **(E–G)** qRT-PCR comparison of the expression *Impdh2* mRNA in oocytes isolated from the primordial, primary, secondary, and Graafian follicles **(E)**; oocytes (OO), cumulus cells (CC), and mural granulosa cells (MGC) isolated from the large antral follicles **(F)**; oocytes at GV- and MII stages, and preimplantation embryos at different developmental stages **(G)**. Bars without letters in common are considered to be significantly different (*P* < 0.05).

Generation of cGMP by NPR2 guanylyl cyclase requires the supply of GTP substrate. As illustrated in Figure 1A, GTP is produced through both the *de novo* biosynthesis pathway with inosine 5′-phosphate (IMP) as the precursor and the salvage pathway in which guanine is converted into GMP by hypoxanthine guanine phosphoribosyltransferase (HPRT). IMP is mainly produced from the *de novo* purine nucleotide biosynthesis, and to a lesser extent, is also converted from hypoxanthine by HPRT via the purine nucleotide salvage pathway. IMP dehydrogenase (IMPDH) is the rate-limiting enzyme in the GTP *de novo* biosynthesis pathway, which catalyzes conversion of IMP to xanthosine 5′-phosphate (XMP), the first committed and rate-limiting step of GTP biosynthesis. XMP is subsequently converted to GMP by the GMP synthetase (GMPS), and GMP is sequentially converted to GTP by GMP kinase and nucleoside diphosphate kinase (Hedstrom, 2009). In line with the essential role of IMPDH in the biosynthesis of guanine nucleotides, IMPDH was found to be crucial for the maintenance of oocyte meiotic arrest. A serial of earlier studies from the Eppig group in the 1990s revealed that inhibitors of IMPDH induced the resumption of meiosis by competent oocytes both *in vivo* and *in vitro* in a way that is independent of the LH, highlighting the importance of IMPDH-mediated biosynthesis of guanine nucleotides in supporting oocyte meiotic arrest maintenance (Downs et al., 1986; Downs and Eppig, 1987; Eppig, 1991). Remarkable, a recent study by the same group demonstrated that IMPDH inhibitors efficiently reverse oocyte meiotic arrest imposed by NPPC and hypoxanthine in cultured COCs *in vitro*. Coincidently, IMPDH inhibitors also reduce the levels of cGMP produced by these COCs (Wigglesworth et al., 2013). This study thus brings the direct connection of IMPDH to the production of cGMP by granulosa cells needed for delivery to oocytes to sustain oocyte meiotic arrest. Interesting, this study also found that the expression of IMPDH in cumulus cells is promoted by oocyte-derived paracrine factors, indicating the fine-tuned regulation of the expression of IMPDH is crucial for oocyte meiotic control (Wigglesworth et al., 2013). The meiotic arrest inputs from granulosa cells are reported to be withdrawn or turned off after the LH surge in order to allow for the reinitiation of oocyte meiosis (Norris et al., 2009; Vaccari et al., 2009; Downs, 2010; Jaffe and Egbert, 2017). How IMPDH is accordingly changed with the induction of oocyte meiotic resumption remains unclear. Furthermore, the dynamic changes in the expression and function of IMPDH in the oocytes also remain unknown. These are the questions that we are trying to address in this study.

There are two isoforms of IMPDH in mammals, IMPDH1 and IMPDH2. These two members share 84% aminio acid sequence identity and have virtually indistinguishable catalytic activity, but their tissue expression patterns and physiological importance differ largely (Collart and Huberman, 1988; Carr et al., 1993; Senda and Natsumeda, 1994; Hager et al., 1995). IMPDH2 is the dominant isoform expressed in most tissues of human, while IMPDH1 is found to be constitutively expressed at low levels in most tissues other than retina, spleen, and resting peripheral blood mononuclear cells (Senda and Natsumeda, 1994; Jain et al., 2004; Bowne et al., 2006). Accordingly, knockout (KO) of *Impdh2* in mice leads to early embryonic lethality, which is in contrast with the *Impdh1*-KO that causes no overt abnormalities except a mild retina defect (Gu et al., 2000; Gu et al., 2003). Therefore, IMPDH2 becomes a more attractive target for the development of immunosuppressive, antiviral, and cancer therapeutics (Sintchak and Nimmesgern, 2000). Most intriguingly, IMPDH2 was found, under certain circumstances that interfere with the guanosine nucleotide homeostasis, to aggregate into a non-membranous filamentous subcellular macrostructure termed “cytoophidium” or “rod and ring” in a variety types of mammalian cells being studied (Liu, 2016; Calise and Chan, 2020). For example, in MCF7 human breast adenocarcinoma cell line, inhibition of IMPDH activity by treatment with mycophenolic acid (MPA), the non-competitive inhibitor of IMPDH, induces the aggregation of IMPDH2 into the rod- and ring- shaped marcrostructure, the first report on the formation of IMPDH2 cytoophidium (Ji et al., 2006). Similar observations were also made later in other types of cancer cell lines, as well as mouse primary cells (Carcamo et al., 2011; Thomas et al., 2012). Although it appears from the earlier reports that formation of IMPDH2 cytoophidia is a consequence of IMPDH2 inhibition or GTP insufficiency, recent studies indicate that formation of IMPDH2 cytoophidia are not an artificial phenomenon of perturbation of guanosine biosynthesis *in vitro*, it occurs under normal physiological conditions and may actively engage in certain biological processes involving purine metabolism. For instance, aggregation of IMPDH2 into cytoophidia were found to occur naturally in mouse embryonic stem cells (ESCs) and T-cell, which is crucial for rapid cell proliferation, and T-cell activation (Carcamo et al., 2011; Calise et al., 2018; Keppeke et al., 2018). Formation of IMPDH2 cytoophidia was also found *in vivo* in mouse pancreatic β cells, which was predicted to correlate with insulin secretion (Chang et al., 2015; Keppeke et al., 2019). However, the biological significance and underlying mechanism of IMPDH2 assembling into cytoophidia remains elusive. Whether or not IMPDH2 also assembles into cytoophidia within the ovaries is not clear.

Given the crucial role of granulosa cell IMPDH in the regulation of oocyte meiotic arrest, oocytes and the associated somatic cells in the ovary hence render an exciting model system for exploring the functional significance of IMPDH cytoophidium formation within the cell. Herein, by examining the dynamic expression and localization of IMPDH2 in the oocytes and granulosa cells throughout the process of oocyte growth and maturation, we show that cytoophidia do form in the oocytes and granulosa cells at particular stages of development, which may contribute to the oocyte acquisition of meiotic and developmental competences and the induction of meiosis reinitiation, respectively.

## Materials and Methods

### Ethics Statement

All animal protocols were approved by the Ethical Committee of Laboratory Animals and the Animal Care and Use Committee of Nanjing Medical University (NJMU), and all experiments were conducted in accordance with the Institutional Guide for the Care and Use of Laboratory Animals.

### Mice

Female ICR mice were used in all experiments. These mice were purchased from the Animal Core Facility of Nanjing Medical University. Adult C57BL/6JXDBA2 (B6D2) F1 male mice were produced at the investigator’s own colony, and were used for the *in vitro* fertilization experiments. All mice were housed in ventilated cages on a 12-h light: 12-h dark cycle at constant temperature (22°C) and controlled humidity.

### Chemicals and Reagents

Unless otherwise stated, all chemicals and reagents were purchased from Sigma, United States.

### Oocytes and Granulosa Cell Isolation, Culture, and Treatments

Female mice were injected with 5 IU eCG (Ningbo Second Hormone Factory) for 46 h to stimulate follicular development. Cumulus-oocyte complexes (COCs) were then isolated from the large antral follicles, and denuded oocytes (DOs) were obtained by stripping off the cumulus cells from these COCs. Mural granulosa cells, cumulus cells, and DOs were then collected as described previously for reverse transcription quantitative realtime PCR (qRT-PCR) and Western Blot (WB) analysis (Su et al., 2006). Oocytes of primordial, primary, and secondary follicles were isolated from ovaries of neonatal female mice at the age of day 3, 6, and 12, respectively, using a protocol as detailed in the previous studies (Su et al., 2012b; Guo et al., 2018). To collect the oocytes at GV, pro-metaphase I (Pro-MI), MI, anaphase I (AI), telophase I (TI), and MII stages, oocytes were cultured in maturation medium, and the samples were collected at the 0-, 4-, 6-, 8-, 10-, and 14-h intervals, respectively. To investigate the earlier changes of IMPDH2 expression in oocytes and COCs after the LH surge, female mice were first primed with 5 IU/mouse of eCG for 48 h, followed by injection with hCG (5 IU/mouse). COCs were then collected at the 0, 2, 4, 6-h intervals following hCG injection by puncturing the large antral follicles with a pair of 26-gauge needles connected to 1ml- syringes. To obtain the oocytes from theses COCs, COCs were first briefly (3–5 min) treated with 1mg/ml hyaluronidase, and cumulus cells were then stripped off oocytes by passing COCs several times through a glass pipette with an inner diameter slightly narrower than the oocyte.

To investigate the effect of inhibition of IMPDH2 on the subcellular localization of IMPDH2 in cumulus cells and the maintenance of meiotic arrest in oocytes, COCs were cultured in medium supplemented with 25 μM NPPC (TOCRIS, Cat #3520) and 100 μM estradiol (E_2_) to maintain oocyte meiotic arrest, and treated with different doses of INPDH inhibitor, mycophenolic acid (MPA), for a period up to 6 h. At different intervals of the culture, assessment of oocyte GVBD and assembly of IMPDH2 cytoophidia were carried out. NPPC and E_2_ were dissolved in distilled water and DMSO at a stock concentration of 25 and 100 mM, respectively. MPA was dissolved in DMSO at a stock concentration of 100 mM. Groups treated with 100 μM DMSO served as controls.

To compare the potential difference of inhibition of IMPDH on IMPDH2 localization and oocyte meiotic maturation in cumulus cell-enclosed oocytes (CEOs) and DOs, COCs, and DOs were cultured in maturation medium, and treated with 100 μM MPA for 14 h. At the end of culture, the extrusion of the first polar body and the meiotic status of the oocytes were assessed. Alternatively, to assess the effect of inhibition of IMPDH on the kinetics of GVBD and the developmental competence of oocytes, DOs were cultured in maturation medium supplemented with 100 μM MPA for up to 14 h. GVBD was scored in a 15- min interval during the initial 2-h period of culture, and *in vitro* fertilization (IVF) was carried out on the matured oocytes at the end of 14-h culture. To assess the effect of inhibition of IMPDH on preimplantation development, oocytes that were matured *in vitro* were first subjected to IVF by incubating with the capacitated sperm for 6 h, and then the sperm were washed off, and the fertilized oocytes were transferred into medium supplemented with 100 μM MPA for further culture. DOs that were treated with only 100 μM DMSO served as controls.

To test the effect of guanosine supplement and inhibition of MTOR pathway on MPA-induced aggregation of IMPDH2 in cumulus cells and meiotic resumption in oocytes, COCs were cultured in NPPC and E_2_ containing medium, and treated with 100 μM MPA, 100 μM MPA + 200 μM guanosine, 1 μM MTOR inhibitor Torin1, or 100 μM MPA + 1μM Torin1 for 14 h. At the end of culture, Oocyte GVBD and cytoophidium assembly were then analyzed. COCs that were treated with only 100 μM DMSO served as controls.

Oocyte collection and culture was carried out in bicarbonate-buffered minimum essential medium with Earle’s salts (Thermo Fisher Scientific Inc., Waltham, MA, United States) supplemented with 75 μg/ml penicillin G, 50 μg/ml streptomycin sulfate, 0.23 mM pyruvate, and 3 mg/ml bovine serum albumin. To prevent the oocyte undergo spontaneous maturation during the collection period, collection medium was supplemented with 2.5 μM milrinone, the specific inhibitor of PDE3A. Cultures were carried out at 37°C in an Eppendorf NewBrunswick Galaxy170R incubator (Hamburg) infused with 5% O_2_, 5% CO_2_ and 90% N_2_.

### *In vitro* Fertilization and Embryo Culture

Oocyte *in vitro* fertilization and embryo culture were carried out according to the procedure as described in the previous study (Eppig and O’Brien, 1996). Briefly, following IVM, mature oocytes with visible first polar bodies were inseminated with the capacitated sperm isolated from BDF1 adult males in the Petri dishes. The formation of pronuclei and 2-cell stage embryos were assessed at 8- and 24- h after IVF, respectively. After 24 h, the 2-cell stage embryos were transferred into KSOM medium and cultured for another 3–5 days, embryo cleavage was then scored during the culture.

### Immunofluorescence Analysis

For immunofluorescence (IF) analysis of ovaries, freshly isolated ovaries were fixed in 4% paraformadehyde solution for 4 h, and embedded in paraffin. Ovarian blocks were sectioned at 5-μm thickness, and processed for IF analysis using the anti-IMPDH2 primary antibody (1:100, Proteintech, #12948-1-AP, Wuhan, China) and Alexa fluor 594-conjugated donkey anti-rabbit secondary antibody (1:200, Thermo Fisher Scientific, Waltham, MA, United States) as described previously (Guo et al., 2016). Oocyte whole mount IF analysis was carried out using the same protocol as previously described (Su et al., 2012a). Briefly, oocytes were first fixed in 4% paraformaldehyde for 30 min at room temperature, followed by washing in PBS containing 0.1% fetal bovine serum (FBS) and permeabilization with 0.5% Triton X-100 in PBS for 20 min at room temperature. The oocytes were then blocked with 10% FBS in PBS for 30 min, and subsequently incubated with primary antibodies (4°C, overnight) and Alexa flour 594-conjugated secondary antibodies (room temperature, 1 h), respectively. After counterstaining with Hoechst 33342 for 10 min to label chromosomes, the oocytes were finally mounted on glass slides in antifade, and subjected to confocal microscopy. Antibodies used were rabbit polyclonal anti-IMPDH2 (1:1,000, Proteintech, #12948-1-AP) and mouse monoclonal FITC-labeled anti-αTubulin (1:600) primary antibodies, and Alexa fluor 594-conjugated donkey anti-rabbit secondary antibody (1:1,000, Thermo Fisher Scientific, Waltham, MA). Imaging was carried out using a LSM 700 confocal laser scanning microscope (Zeiss, Oberkochen, Germany) with the identical settings for the control and treated groups within the same experiments.

### Western Blot Analysis

Samples of mural granulosa cells (MGCs), cumulus cells (CCs), oocytes, and preimplantation embryos were collected and processed for Western Blot (WB) analysis as described previously (Su et al., 2001, 2010). Briefly, samples were collected and lysed in 2 × Laemmli sample buffer, and heated at 108°C for 5 min before electrophoresis. The denatured protein samples were then loaded in the 10% polyacrylamide gel, resoled by the SDS-PAGE and transferred onto polyvinylidene difluoride (PVDF) membranes for protein detection as detailed in the previous study (Su et al., 2001, 2010). IMPDH2 was detected by anti-IMPDH2 primary antibody (1:1,000, Proteintech, #12948-1-AP). The expression of β-actin (ACTB) detected by anti-β-actin antibody (1:2,000, A1978) served as internal control of each sample. Quantification of the WB data was carried out by Image J according to the instructions provided by the manufacturer.

### Reverse-Transcription and Real-Time PCR Analysis

Samples of oocytes, CCs, MGCs, and preimplantation embryos were collected in RLT buffer, and stored at −80°C until RNA extraction. Total RNA was extracted and reversed transcribed using the RNeasy Micro Kit (Qiagen) and QuantiTect Reverse Transcription Kit (Qiagen), respectively. The SYBR Green-based realtime PCR analysis was then carried out on these cDNAs using the primer pairs for *Impdh2* and *Rpl19*. The sequences of these primary pairs are: *Impdh*-Forward 5′-GACTTACTGGCCCTTGCTGG-3′, *Impdh*-Reverse 5′-CCACAGGCCAACACTTCCT-3′; *Rpl19-*Forward 5′-GGAAAAAGAAGGTCTGGTTGGA-3′, *Rpl19-*Reverse 5′-GGCGGTCAATCTTCTTGGATT-3′. The relative fold changes in the mRNA levels of *Impdh2* were calculated using the method of 2^–ΔΔCt^ as described previously (Su et al., 2007), with *Rpl19* served as an internal control.

### Statistics

All experiments were performed independently for at least three times, and data are presented as Mean ± SEM. Statistical analyses were conducted using Graphpad Prism software (Graphpad software, Inc., La Jolla, CA, United States). For experiments with only two groups, Student’s *t*-test was applied to determine the differences between the groups. For experiments containing more than two groups, differences between groups were compared by one-way ANOVA followed by Tukey’s Honestly Significant Difference (HSD) test. Differences with a *P*-value less than 0.05 are defined as significant.

## Results

### Dynamic Changes in the Expression of *Impdh2* in Oocytes, Granulosa Cells, and Preimplantation Embryos

Quantitative RT-PCR and Western Blot analyses were performed to investigate the dynamic changes in the expression of *Impdh2* in oocytes at various developmental stages, cumulus and mural granulosa cells of large antral follicles, and preimplantation embryos (Figure 1). IMPDH2 protein was expressed at constant levels in oocytes during the entire process of development and maturation (Figures 1B,D). However, at mRNA levels, a different pattern of *Impdh2* expression was observed during oocyte development and maturation (Figures 1E,G). The levels remained unchanged during the transition from the non-growing primordial follicle stage to the growing primary and secondary follicle stages. A sharp reduction took place when the oocytes reached the fully grown stage in the Graafian follicles (Figure 1E), and a further dramatic decrease was observed when the oocytes matured to the metaphase II (M II) stage (Figure 1G). In the Graafian follicles, both *Impdh2* protein and mRNA were found to be expressed at relatively lower levels in the oocytes than the cumulus and mural granulosa cells, with the highest levels detected in cumulus cells (Figures 1C,F). The levels of IMPDH2 protein were not evidently changed in the pronuclear and 2-cell stage embryos after fertilization, but increased sharply after the second round of embryonic cleavage (Figure 1D). At the mRNA levels, *Impdh2* was decreased to the barely detectable levels at the pronuclear stage, and then restarted to be expressed at 2-cell stage, with the most robust expression observed at 4-cell stage and onward (Figure 1G).

### Dynamic Changes in the Subcellular Localization of IMPDH2 in Oocytes and Preimplantation Embryos

**FIGURE 2:**
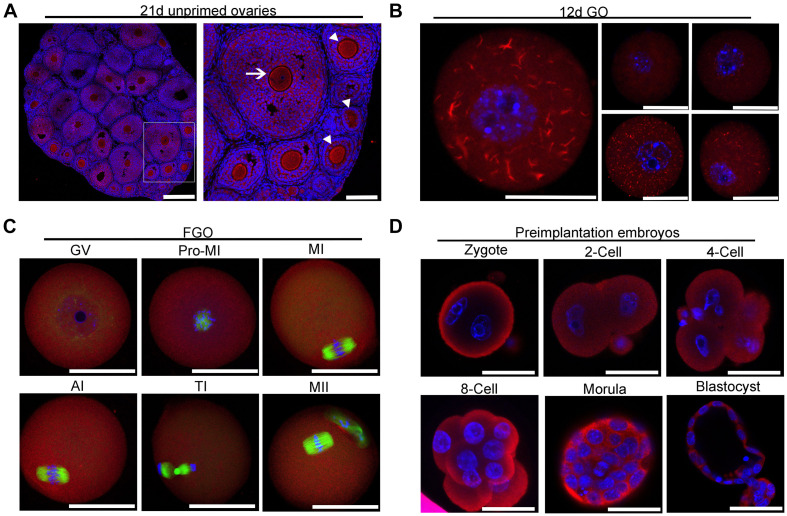
Localization of IMPDH2 in the ovary, oocytes and preimplantation embryos. **(A)** IF staining of IMPDH2 protein in the ovary isolated from 21 day-old female mice that were not primed with eCG. The arrow indicates the oocyte within a small antral follicle, arrowheads point to oocyte in early stage growing follicles. In this and the following all figures, IMPDH2 is stained in red, DNA is counterstained in blue. Scale bars, except those in B that represent 20 μm, are 50 μm. **(B–D)** Whole mount IF staining of IMPDH2 in the growing oocytes isolated from the ovaries of 12 d-old female mice **(B)**; fully grown oocytes (FGO) at different maturation (GV, Pro-MI, MI, AI, TI, and MII) stages **(C)**; and preimplantation embryos at different developmental (PN, 2-Cell, 4-Cell, 8-Cell, Morula, and Blastocyst) stages **(D)**. In **(C)**, α-tubulin is stained in green.

To investigate the intraovarian localization of IMPDH2, immunofluorescence staining was performed on 21 day-old ovarian sections. As shown in Figure 2A, IMPDH2 was detected in both the oocytes and granulosa cells of all stages of follicles. In the early stage growing follicles, a relatively stronger signal of IMPDH2 staining was observed in oocytes than the companion granulosa cells, and this difference was diminished gradually as the secondary follicles growing larger. No apparent difference in the IF staining of IMPDH2 was observed between the oocytes and granulosa cells in the small antral follicles. To further examine if there is any specific patterns of subcellular localization of IMPDH2 during the processes of oocyte and preimplantaion embryo development, whole mount IF staining of IMPDH2 was conducted on the growing and fully grown oocytes (Figures 2B,C), as well as preimplantation embryos (Figure 2D). In the growing oocytes isolated from the ovarian follicles of 12-d old mice, IMPDH2 was found to be localized in both the cytoplasm and nucleus, and formed aggregates with variable size and shape resembling those “cytoophidia” or “rods and rings” subcellular macrostructure reported previously in other types of cells (Figure 2B). However, complete dissolution of the IMPDH2 aggregates took place when the oocytes reached the fully grown stage, which resulted in the even distribution of IMPDH2 inside the oocytes during the process of meiotic maturation (Figure 2C). No apparent aggregation of IMPDH2 was found either in the preimplantation embryos (Figure 2D). Except in the pronuclear stage embryos where more enrichment of IMPDH2 was found at the subcortical region, IMPDH2 was ubiquitously distributed in the cytoplasm of the rest stages of preimplantation embryos.

### Aggregation of IMPDH2 Into Cytoophidium and Reduction of IMPDH2 Expression in Granulosa Cells of Preovulatory Follicles

**FIGURE 3:**
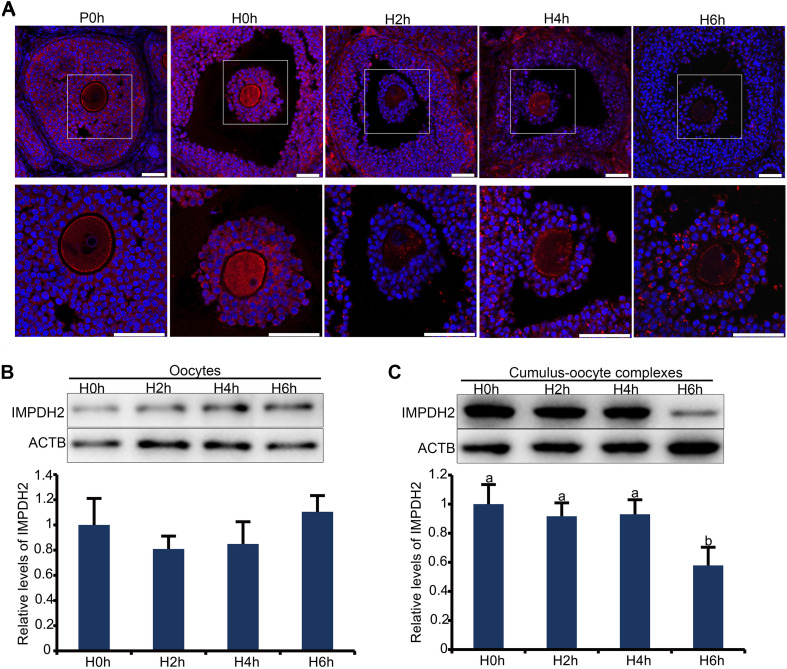
Dynamic changes in the localization and expression of IMPDH2 protein in the oocyte and granulosa cells of the preovulatory follicles after *in vivo* administration of hCG to the mice. **(A)** IF staining of IMPDH2 protein in the ovary isolated from female mice that were not primed with eCG (P0h), primed with eCG for 48h but no hCG (H0h), and primed with eCG for 48 h followed by hCG for 2h (H2h), 4h (H4h), and 6h (H6h), respectively. Magnified view of the boxed area in the top panels is shown at the bottom. IMPDH2 is stained in red, and DNA is counterstained in blue. Scale bars represent 100 μm. **(B**,**C)** WB detection of the expression of IMPDH2 protein in the oocytes and cumulus-oocyte complexes isolated from ovarian follicles of female mice that were primed with eCG for 48 h but no hCG (H0h), and primed with eCG for 48 h followed by hCG for 2h (H2h), 4h (H4h), and 6h (H6h), respectively. Quantification of the WB results is shown in the bottom bar graphs, bars without letters in common are considered significantly different (*P* < 0.05).

In the large antral follicles, IMPDH in granulosa cells was reported to be responsible for the maintenance of oocyte meiotic arrest before the appearance of the preovulatory LH surge. It is therefore plausible to speculate that the expression and/or activity of IMPDH in granulosa cells must be downregulated after the surge of preovulatory LH. We tested this possibility by assessing changes in the localization and expression of IMPDH2 in granulosa cells after *in vivo* administration of gonadotropins. IF staining of the ovarian sections revealed that the pattern of localization of IMPDH2 in granulosa cells was not changed after eCG priming, but the IF staining signal was getting brighter, which is indicative of the increase in the levels of expression. The increase of IMPDH2 expression in granulosa cells of large antral follicle after eCG priming could contribute to the maintenance of oocyte meiotic arrest by production of sufficient amount of GTP substrate for NPPC/NPR2. Evident changes in the localization and expression of IMPDH2 were observed in granulosa cells after administration of hCG. IMPDH2 started to nucleate and form small aggregates in certain granulosa cells 2 h after hCG injection, which was intensified over time. Massive nucleation of IMPDH2 was observed in most, if not all, of the granulosa cells 4–6 h after hCG injection, and the aggregates got elongated resembling the phase two cytoophidium as illustrated previously. Simultaneously, the IF staining signal of IMPDH2 diminished in the granulosa cells following hCG administration, with the most dramatic decrease observed 6 h after hCG injection (Figure 3A). To further verify the changes in the levels of IMPDH2, WB analysis was performed on oocytes and COCs isolated from the large antral follicles at different intervals after hCG administration. The levels of IMPDH2 remained constant in the oocytes at all three timepoints examined (Figure 3B), which was in contrast with the COCs where a gradual decrease was detected. Significant reduction in the levels of IMPDH2 was observed in COCs 6 h after hCG injection (Figure 3C).

### Inhibition of IMPDH2 Activity in COCs Induced the Coincident Formation of Cytoophidia in Cumulus Cells and Reversal of Oocyte Meiotic Arrest Maintained by NPPC in Culture

**FIGURE 4:**
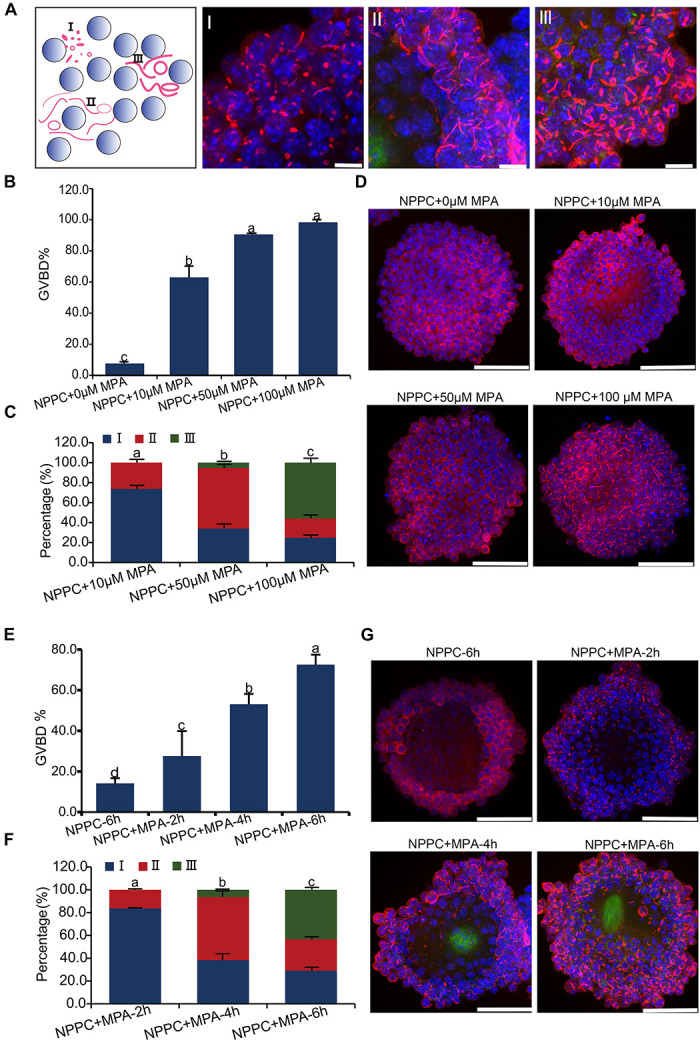
Inhibition of IMPDH activity in COCs cultured in NPPC-supplemented medium induces reversal of oocyte meiotic arrest and aggregation of IMPDH2 into cytoophidia in cumulus cells. **(A)** Schematic illustration (far left panel) and representative images of the three types of IMPDH2 cytoophium classified based on the shape and size (length and thickness) of the aggregates. **(B–D)** Effects of treating COCs with different doses (0, 10, 50, 100 μM) of IMPDH inhibitor MPA on oocyte meiotic resumption **(B)** and cumulus cell IMPDH2 aggregation **(C,D)**. **(E,F)** Effects of treating COCs with 100 μM- MPA for different time (2, 4, 6 h) on oocyte meiotic resumption **(E)** and cumulus cell IMPDH2 aggregation **(F,G)**. COCs not treated with MPA and cultured for 6 h served as control. IMPDH2 and DNA are stained in red and blue, respectively. Scale bars: 50 μm. Bars without letters in common are considered to be significantly different (*P* < 0.05).

Nucleation and assembly into cytoophidia is a common feature for IMPDH2 when its activity was inhibited *in vitro* in a variety of cell types (Liu, 2016; Calise and Chan, 2020). We wonder whether the formation of cytoophidia by IMPDH2 is correlated with the reduction in the activity of IMPDH2 and induction of oocyte meiotic resumption. To address this question, we assessed the changes of IMPDH2 localization upon inhibition of its activity in COCs by mycophenolic acid (MPA), an ancient inhibitor of IMPDH, *in vitro*. To mimic the *in vivo* physiological conditions, COCs were cultured in the medium supplemented with NPPC, the physiological oocyte maturation inhibitor existed in the follicular fluids, to maintain oocyte meiotic arrest. Three major types of cytoophidium were observed in the COCs treated with various doses of MPA. As illustrated in Figure 4A, for the ease of description, we designated these three major types of cytoophidia as “Type I,” “Type II,” and “Type III,” respectively. Type I represents the early phase of cytoophidium assembly, with the major shape of small rings. Type II is at the intermediate phase, with the thin-rod shaped cytoophidia prevalent. Type III represents the most advanced phase of cytoophidium assembly, with most of the cytoophidia in the shape of big ring and large rod.

When COCs were cultured in medium supplemented with 25-μM NPPC for 6 h, only 7.7% of oocytes resumed meiosis, and no apparent assembly of IMPDH2 into cytoophidia was observed in cumulus cells (Figures 4B,D). When MPA was added into the culture medium, it dose-dependently induced the resumption of meiosis. Under the treatment of 10 μM MPA, the rate of GVBD was already reached 63%. GVBD rate was increased to 90.6% when COCs were treated with 50 μM MPA, and the maximum induction of GVBD (98.3%) was achieved by 100 μM MPA treatment (Figure 4B). Coincident with the resumption of meiosis in oocytes, evident assembly of IMPDH2 into cytoophidia was detected in cumulus cells surrounding the oocyte (Figures 4C,D). The size and shape of the IMPDH2 aggregates appeared to be dependent on the doses of MPA applied. Under 10 μM MPA treatment, most (73.7%) of the cytoophidia were type I, and a small portion (26.3%) were type II. With the treatment of 50-μM MPA, the assembly of cytoophidia was driven into the intermediate phases, with type II cytoophidia prevalent (60.8%). Cytoophidia assembly in cumulus cells reached the most advanced phase after COCs were treated with 100 μM of MPA, with the major type of cytoophidia was type III (55.9%).

Moreover, the type of cytoophidia formed by IMPDH2 is dependent on the duration of MPA treatment, and is tightly associated with the degree of oocyte resumption of meiosis. As shown in Figures 4E–G, when COCs were treated with 100 μM of MPA for only 2 h, most (83.7%) of the cytoophidia formed in the cumulus cells were type I, and there was only 27.6% of the oocytes underwent GVBD. After COCs were treated with 100 μM of MPA for 4 h, type II cytoophidia became prevalent in the cumulus cells and the oocyte GVBD rate increased to 53.1%. By 6 h of MPA treatment, a large proposition (43.4%) of the cytoophidia were transformed to type III in cumulus cells, and oocyte GVBD reached 72.6%.

### Effect of Inhibition of IMPDH2 Activity on Oocyte and Preimplantation Embryo Development

**FIGURE 5:**
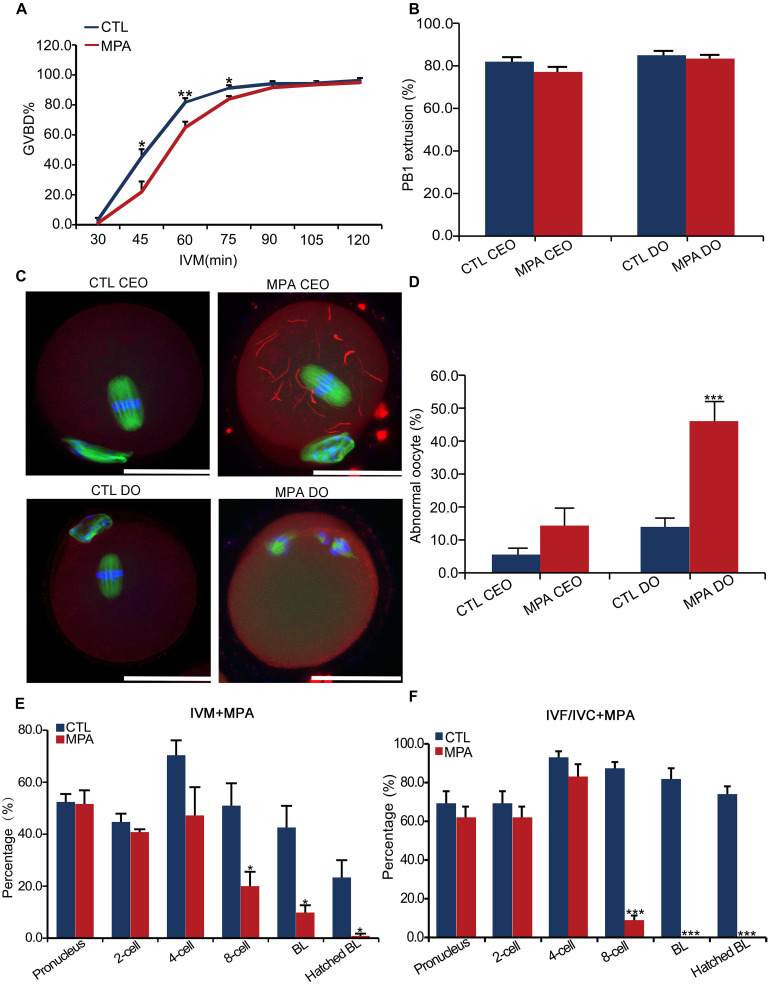
Effects of inhibition of IMPDH activity on oocyte IMPDH2 aggregation and the development of oocyte and preimplantation embryos. **(A)** Effects of 100 μM- MPA treatment on the kinetics of GVBD in DOs *in vitro*. *P < 0.05, **P < 0.01, compared with the control (CTL) at the ame timepoint. **(B)** Effects of 100 μM– MPA treatment on PB1 extrusion in cumulus-enclosed oocytes (CEO) and DOs *in vitro*. **(C,D)** Effects of 100 μM- MPA treatment on IMPDH2 aggregation and the maturation into normal MII oocytes in CEO and DOs *in vitro*. Representative micrographs of staining of IMPDH2, α-tubulin, and chromosomes are shown in **(C)**, while quantification of the rate of matured oocytes with abnormal spindles and misaligned chromosomes is shown in **(D)**. Scale bars: 50 μm. ***P < 0.001, compared with the CTL. **(E)** Effects of treating the DOs with 100 μM- MPA during the process of maturation on the subsequent fertilization and preimplantation development. *P < 0.05, compared with the CTL at the same stage of embryos. **(E)** Effects of treatment with 100 μM- MPA on the development of the preimplantation embryos. ***P < 0.001, compared with the CTL at the same stage of embryos.

The expression of IMPDH2 in oocytes and preimplantation embryos suggests that it may play a role during oocyte maturation and preimplantation development. We tested this possibility by inhibiting the activity of IMPDH2 with 100 μM of MPA during the process of oocyte and embryo culture. Treatment with MPA only slightly delayed GVBD in cultured denuded oocytes (DO) but not cumulus-enclosed oocyte (CEO) within the first 90 min of *in vitro* maturation (IVM) (Figure 5A). It did not affect the extrusion of the first polar body (PBE) in either DO or CEO (Figure 5B). Interestingly, MPA treatment induced the formation of cytoophidia by IMPDH2 in CEOs but not DOs (Figure 5C), which is in strike contrast to its effects on the quality of CEOs and DOs (Figures 5D,E). Deleterious effects on oocyte quality were only observed in DOs but not CEOs that were matured in the presence of MPA. Treatment with MPA during the process of DO-IVM significantly increased the rate of abnormal oocytes having disorganized metaphase II (MII) spindles and misaligned chromosomes (Figure 5D). After *in vitro* fertilization (IVF) of these DOs matured in the presence of MPA, the formation of pronuclei and the initial two rounds of embryo cleavage were essentially normal. However, the development of the 4-cell stage embryos toward hatched blastocysts was dramatically inhibited, few of them developed into blastocyst (Figure 5E). Such deleterious effect on the potential of preimplantation development was not observed in CEOs matured in the presence of the same dose of MPA (data not shown).

In accord with the robust upregulation of IMPDH2 mRNA and proteins in 4–8 cell stages of embryos, treatment with 100 μM of MPA during the *in vitro* culture of the fertilized eggs severely interrupted the development of the embryos beyond the 4-cell stage, with no blastocysts formed (Figure 5F).

### Cytoophidium Assembly and Oocyte Meiotic Arrest in COCs Are Affected by Guanylyl Metabolites and MTOR Signaling

**FIGURE 6:**
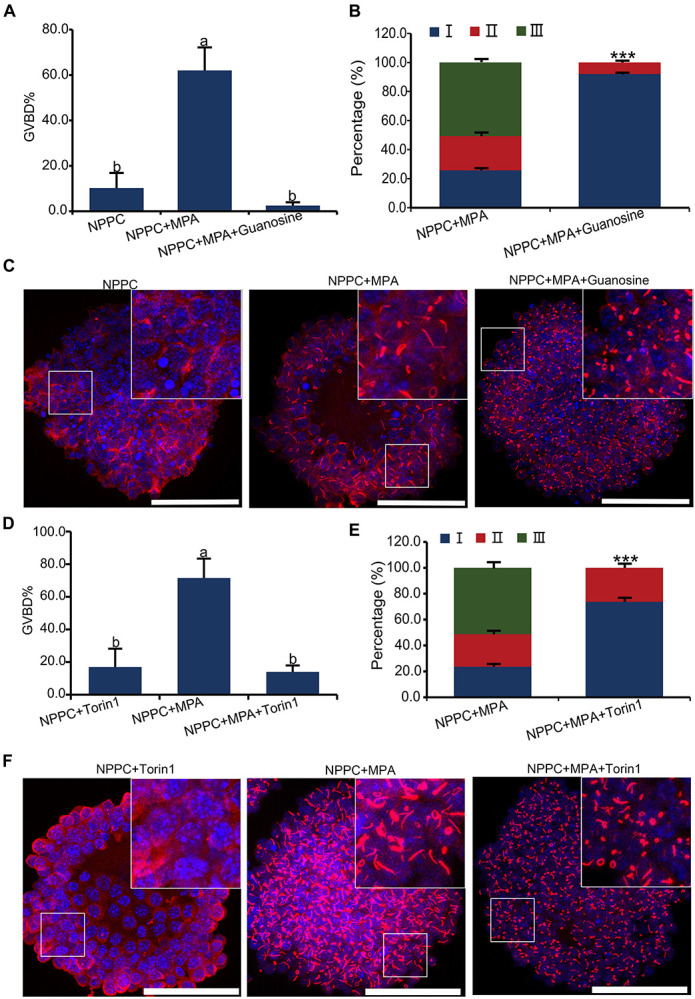
Effects of guanosine supplementation and MTOR inhibition on MPA-induced reversal of oocyte meiotic arrest and aggregation of IMPDH2 into cytoophidia in cumulus cells. **(A–C)** Treating COCs with 200 nM- guanosine inhibits MPA-induced oocyte GVBD **(A)** and formation of IMPDH2 cyoophidia in cumulus cells **(B,C)**. **(D–F)** Treating COCs with MTOR inhibitor Torin1 (1 μM) inhibits MPA-induced oocyte GVBD **(D)** and formation of IMPDH2 cyoophidia in cumulus cells **(E,F)**. Scale bars: 50 μm. Bars without letters in common are considered significantly different (*P* < 0.05). ***P < 0.001.

Aggregation and activation of IMPDH2 can be affected by the availability of its guanylyl product, as well as the activation of the MTOR pathway in T cells (Duong-Ly et al., 2018). We tested whether this could be the same case as well in COCs. As illustrated in Figure 1A, guanosine can be cleaved into guanine, which is then able to be enzymatically joined with PRPP by HPRT to convert into GMP. Therefore, guanosine was able to bypass the inhibitory effect of MPA on IMPDH and GTP production, and abrogate the reversal effect of MPA on oocyte meiotic arrest maintained by NPPC (Wigglesworth et al., 2013). We wondered whether guanosine could also interfere with the effect of MPA on the assembly of IMPDH2 into cytoophidia in COCs. To address this question, we treated the COCs that were cultured in NPPC-supplemented medium with 200-nM guanosine together with 100 μM of MPA, and assessed the ability of IMPDH2 to assemble into cytoophidia. Unsurprisingly, oocyte GVBD induced by MPA was completely suppressed by guanosine (Figure 6A). Importantly, we found that guanosine profoundly impaired the formation of cytoophidia induced by MPA, most (92.1%) of the cytoophidia formed were at the early stage of assembly, belonging to type I (Figures 6B,C).

To investigate the involvement of MTOR signaling in the assembly of IMPDH2 cytoophidium in cumulus cells and the maintenance of oocyte meiotic arrest, MTOR pathway in COCs was inhibited by treatment with Torin1, a specific inhibitor of MTOR. We observed that the MPA-induced oocyte GVBD and the assembly of IMPDH2 cytoophidia in cumulus cells were near completely abrogated by treatment with 1 μM Torin1 (Figures 6D–F). The rate of oocyte GVBD in COCs treated with 1-μM Torin1 + 100-μM MPA was only ∼15%, which was significantly lower than that (∼75%) in COCs treated with only 100-μM MPA. Only type I cytoophidia were formed in cumulus cells of COCs treated with Torin1 + MPA. No significant alteration of oocyte GVBD was observed in COCs that were only treated with 1-μM Torin1, neither did the assembly of IMPDH2, as compared with the control group COCs cultured in medium only supplemented with NPPC (Figure 6A).

## Discussion

In this study, we systematically examined the spacial-temporal expression and distribution of IMPDH2 during the processes of oocyte and follicle growth, oocyte maturation and preimplataion development, and revealed the unique mechanisms that control the dynamic changes in the expression of IMPDH in oocytes, granulosa cells, and preimplantation embryos. We also observed the aggregation of IMPDH2 into cytoophidium both *in vivo* and *in vitro* in the oocytes and granulosa cells at particular stages of development, and revealed that these IMPDH2 aggregation events coordinate with the oocyte acquisition of meiotic and developmental competences and the induction of oocyte meiosis reinitiation. These data therefore unraveled the complex regulation of IMPDH expression and activity in the oocyte and granulosa cells, and demonstrated that the fine-tuned regulation of guanine nucleotide biosynthesis is crucial for the control of oocyte growth and maturation.

We found that both the protein and mRNA of IMPDH2 are expressed in the oocyte and embryo at all the stages examined, but the pattern of changes across different developmental stages differs largely between them. Specifically, IMPDH2 protein was maintained at a constant level in the oocytes of all stages of follicles. But the levels of IMPDH2 mRNA in oocytes were only maintained constant up to the stage of secondary follicles, and an abrupt decrease was observed in the FGOs of Graafian follicles. Given that oocytes undergo global transcriptional silencing as they reach fully grown, the dramatic reduction in the levels of *Impdh2* mRNA could be the result of the transcriptional shutdown and the rapid turnover of the mRNA. Indeed, recent studies indicate that active degradation of certain number of mRNAs exists in the oocytes at the germinal vesicle stage (Morgan et al., 2017), and work from our laboratory demonstrates that MARF1 (meiosis regulator and mRNA stability factor 1) is an oocyte-specific RNase participating in this particular mRNA degradation process (Yao et al., 2018). *Impdh2* mRNA in the FGOs could be degraded through this process. It is well known that oocytes possess a unique gene expression program, through which most maternal mRNAs are expressed during the oocyte growth phase, and then become dormant and stored for later use (Svoboda et al., 2015; Schultz et al., 2018). However, as the oocyte reaching fully grown, *Impdh2* mRNA becomes extremely instable while the protein maintains relatively unchanged, which makes *Impdh2* mRNA unlikely to be such a type of dormant maternal message.

After oocyte meiotic resumption and fertilization, the mRNA of *Impdh2* becomes even more unstable, and enters the same path as most maternal mRNAs do: undergoing two waves of dramatic degradation. Expression of *Impdh2* mRNA restarts at 2-cell stage embryos when the major zygotic genome activation take places. In contrast to what happens with the mRNA, IMPDH2 protein is expressed at a constant level during oocytes maturation, and this level remains unchanged after fertilization until the embryos reaching the 4-cell stage. This result thus indicates that the mRNA decay-coupled active translation of *Impdh2* take places at these particular stages of oocyte and embryo development, and the IMPDH2 protein stays very stable as well. Starting from the 4-cell stage, both the protein and mRNA of *Impdh2* are boosted as the result of embryonic genome activation and to meet with the demand of fast embryo cleavage. These data therefore indicate that the expression of *Impdh2* in oocytes and preimplantation embryos is regulated through a distinct mechanism involving post-transcriptional and translational control, which differs from the commonly known transcriptional control mechanism in somatic cells (Hedstrom, 2009).

In accord with the stable expression of IMPDH2 protein in oocytes and preimplantation embryos, we observed that inhibition of oocyte IMPDH2 activity during the process of *in vitro* maturation significantly compromised the quality of the resulting eggs. The oocytes matured in the presence of MPA displayed abnormal spindles and misaligned chromosomes, and after IVF, the ability of the resulted embryos to develop beyond the 4-cell stage was markedly reduced. This suggests that oocyte expressed IMPDH2 is crucial for promoting both the nuclear and cytoplasmic maturation of the oocyte. Considering the fact that there is no transcription and DNA replication activity in the fully grown oocytes (Eppig et al., 2004), the meiotic and developmental defects observed after inhibition of oocyte IMPDH2 activity is unlikely caused by disturbing the mRNA or DNA synthesis, but rather by impairing the other important functions of the guanine nucleotide products. Interestingly, inhibition of IMPDH2 activity in the embryos also interrupted the embryonic development beyond the 4-cell stage, thus suggesting that embryonic development after this particular stage requires both the maternal and embryonic expressed IMPDH2 proteins.

Recent studies suggest that assembly of IMPDH2 into the filamentous cytoophidium structure provides a new means for the regulation of IMPDH2 (Johnson and Kollman, 2020). Through the assembly of cytoophidium, cells modulate IMPDH activity to balance levels of product and substrate in response to metabolic demand (Liu, 2016; Calise and Chan, 2020; Johnson and Kollman, 2020). The activity and self-assembly of IMPDH2 into cytoophidium is determined by the intracellular levels of IMP and GTP. According to the recent study by Johnson and Kollman, under conditions with high nucleotides demand, such as in proliferating cells, IMPDH2 assembles into cytoophidium and becomes resistant to GTP inhibition. This allows IMPDH activity to be maintained at relatively high levels to produce sufficient GTP for supporting cell proliferation. In line with this hypothesis, we found in this study that prominent cytoophidia are formed in the growing oocyte of secondary follicles, but not in the FGOs, or the preimplantation embryos. Growing oocytes are well known to be at the most active stage of oocyte development, which requires ample supply of nutrients and metabolites to support macromolecule (e.g., protein and mRNA) synthesis and organelle (e.g., ribosome) assembly, two facets of the cytoplasmic maturation process (Eppig et al., 2004). In this scenario, growing oocytes could resemble the proliferating cells, which have high demands for guanine nucleotides. Assembly of IMPDH2 into cytoophidium will render high IMPDH2 activity to the growing oocytes to produce the GTPs required for fast growth of the oocytes.

We also found the formation of IMPDH2 cytoophidum in granulosa cells of the preovulatory follicles after the administration of hCG *in vivo*. The formation of cytoophidium in granulosa cells starts 2 h after hCG injection, which coincides with the reinitiation of oocyte first meiosis (Su et al., 2002; Hsieh et al., 2007). Interestingly, as a consequence of the inhibition of NPR2 activity and activation of cGMP-phosphodiesterase PDE5, rapid decrease in the levels of cGMP following hCG injection has been reported to take place in the granulosa cells of preovulatory follicles before the oocyte meiotic resumption (Egbert et al., 2014, 2016; Shuhaibar et al., 2015, 2016). Based on these reports, it is plausible to speculate that GTP levels could be also decreased in the granulosa cells of the preovulatory follicles as a feedback of the rapid cGMP reduction, which then induces the formation of IMPDH2 cytoophidium in the granulosa cells.

Nevertheless, one cannot rule out the possibility that GTP reduction and IMPDH2 cytoopidium formation is a direct effect of the LH signaling independent of the changes of NPR2 and cGMP levels. In this regard, IMPDH2 cytoopidium formation could be a prerequisite for the induction of oocyte meiotic resumption by the LH surge. Since granulosa cells cease dividing and enter the phase of terminal differentiation after the LH surge, the mechanism for the formation of the IMPDH2 cytoophidium in the preovulatory granulosa cells is apparently different from what is proposed for the proliferating cells. Notably, we also found the gradual decrease of the levels of IMPDH2 protein in the cumulus cells after hCG injection. This could be a reflection of the overall decline in the consumption of GTP by cumulus cells, and to be part of the changes required to accommodate with the remarkable remodeling process of the cumulus cells, i.e., cumulus expansion. Interestingly, a recent study by the Liu laboratory demonstrates that knockdown of IMPDH2 in Hela cells induces the assembly of IMPDH2 into cytoophidium (Keppeke et al., 2018). It is therefore plausible to speculate that the formation of IMPDH2 cytoopidium observed here in cumulus cells of the preovulatory follicles could be a result of the reduction of IMPDH2 protein.

We further confirmed the association of IMPDH2 cytoopidium formation with the induction of oocyte meiotic resumption *in vitro* in a culture system that mimics the meiotic arrest conditions *in vivo*. In this model, COCs are cultured in the medium supplemented with NPPC to maintain oocyte meiotic arrest, and the resumption of meiosis is induced by treating the COCs with IMPDH inhibitor, MPA (Wigglesworth et al., 2013). We observed that coincident with the resumption of oocyte meiosis, MPA treatment induces remarkable changes in the assembly of IMPDH2 in the cumulus cells that cumulates in the formation of typical mature-form cytoophidum. MPA is a reversible non-competitive inhibitor of IMPDH, which functions through occupying the NAD^+^ cofactor binding site of the catalytic domain of IMPDH. MPA-induced aggregation of IMPDH2 into cytoophidium has been reported in a variety type of cells, but the functional relevance is largely unclear (Liu, 2016; Calise and Chan, 2020). The simultaneous occurrence of oocyte meiotic resumption and cumulus cell IMPDH2 cytoopidium formation in COCs after MPA treatment is intriguing. We do not know at this moment whether or not these two events are functional linked. Nevertheless, they both might be simply attributed by the same cause, i.e., the reduction of GTP in cumulus cells. Interestingly, as reported in T-cells (Duong-Ly et al., 2018), MPA-induced assembly of IMPDH2 cytoophidium in cumulus cells is prevented by guanosine supplement and MTOR inhibitors. This suggests that the formation of IMPDH2 cytoopidium observed here in cumulus cells is probably also a response to lower levels of guanine nucleotides. The interruption of cytoophidium formation upon MTOR inhibition could be caused by a different mechanism, possible by interference with the purine *de novo* synthesis pathway, which resulted in lower levels of IMP in cumulus cells.

Taken together, mammalian oocytes are a specific type of highly differentiated cell, with the development controlled by a unique program that is distinct from somatic cells (Eppig et al., 2004; Schultz et al., 2018). Oocyte growth and maturation require the intimate cooperation with the companion granulosa cells and a fine tuned metabolic program (Eppig, 2001; Su et al., 2009; Clarke, 2017). The observations made here in this study may shed new light on understanding the metabolic control of oocyte growth and maturation.

## Data Availability Statement

The raw data supporting the conclusions of this article will be made available by the authors, without undue reservation.

## Ethics Statement

The animal study was reviewed and approved by the Ethical Committee of Laboratory Animals and the Animal Care and Use Committee of Nanjing Medical University (NJMU).

## Author Contributions

Y-QS and SN conceived the study. SN, TZ, CZ, ML, XH, LY, HL, and LS performed the research. SN and Y-QS analyzed the data and wrote the manuscript. All authors contributed to the article and approved the submitted version.

## Conflict of Interest

The authors declare that the research was conducted in the absence of any commercial or financial relationships that could be construed as a potential conflict of interest.
